# A single center experience of transfusion related adverse reaction in pediatric patients with malignant solid and hematological tumors

**DOI:** 10.3389/fped.2026.1724454

**Published:** 2026-04-10

**Authors:** Gan Shan, Chen Ping, Wang Yang, Yang Liuchun, Chen Shu, Hu Hongbing, Duan Ling

**Affiliations:** Department of Blood Transfusion, Wuhan Children’s Hospital (Wuhan Maternal and Child Healthcare Hospital), Tongji Medical College, Huazhong University of Science & Technology, Wuhan, Hubei, China

**Keywords:** haemovigilance, pediatric oncology, platelet transfusion, prophylactic premedication, transfusion management, transfusion related adverse reaction

## Abstract

**Background:**

This study aimed to analyze the clinical characteristics and rate of transfusion related adverse reactions (TRARs) in pediatric patients with malignant solid and hematological tumors.

**Materials and methods:**

A retrospective analysis was conducted on 34,195 blood transfusions (RBC: 41.0%; PLT: 40.7%; FFP: 16.0%; cryoprecipitate: 2.3%) among 9,129 pediatric patients. TRARs were classified and severity graded according to the Chinese Haemovigilance Network criteria. Statistical comparisons were made between serious (*n* = 37) and non-serious (*n* = 194) TRAR cases.

**Results:**

The overall TRAR rate was 680/100,000 (231/34,195), with PLT-associated TRARs being most frequent (1,250/100,000, 174/13,915). Allergic transfusion reactions (ATRs) dominated (95.7%, 221/231), followed by febrile non-hemolytic reactions (FNHTRs, 3.5%, 8/231). Serious TRARs (16.0%, 37/231) primarily involved ATRs (91.9%, 34/37), presenting with respiratory/gastrointestinal symptoms (41.2%) or anaphylactic shock (14.7%). After adjustment for multiple comparisons using the Bonferroni correction, prophylactic premedication was significantly associated with the occurrence of serious TRARs (*p* < 0.001).

**Conclusion:**

Pediatric patients with malignant solid and hematological tumors exhibit a high TRAR incidence, particularly with PLT transfusions. ATRs constitute the majority, underscoring the need for vigilant monitoring and evidence-based prophylaxis. The inefficacy of routine premedication raises important questions about tailored transfusion strategies. Further research is warranted to optimize platelet product selection and TRAR management protocols in this vulnerable population.

## Introduction

Advances in transfusion medicine practices have made blood transfusion a relatively safe therapeutic modality. However, transfusion related adverse reactions (TRARs) can occur and vary in severity from minor to life-threatening (1). Potentially life-threatening TRARs were observed in approximately 1% of blood transfusions (2, 3).

Given the importance of minimizing transfusion risks, many countries have established national haemovigilance networks to monitor TRARs. In the United States, data from the National Healthcare Safety Network (NHSN) from 2013 to 2018 reported over 18,000 TRARs among 8.34 million transfusion (220/100,000) (4). Similarly, the Chinese Haemovigilance Network (CHN) analyzed reports from 87 facilities between 2018 and 2020, providing valuable national-level data with the TRAR rate of 70/100,000 transfusions (2,348/3,375,301) (5). Understanding TRARs in the pediatric population presents unique challenges. Children are not simply small adults; their distinct physiology, pathology, and immunological development necessitate specific consideration in transfusion practice (6, 7). Reported TRAR rates in the general pediatric population vary between approximately 0.54% and 1.3% per transfused blood product (8–10). Children with cancer represent a distinct population due to their underlying disease biology, intensive treatment regimens, and the consequential alterations in their immune and inflammatory status. Contemporary research in pediatric hematologic malignancies, for instance, highlights the profound and distinct immunological landscape shaped by the disease itself, which can influence treatment response and complications (11). Furthermore, the exploration of specific receptor pathways and molecular mechanisms in cancers underscores the complexity of host-tumor interactions and their systemic effects (12). While these studies advance our understanding of disease pathogenesis and therapeutic targets. They also implicitly underscore that the clinical management of these patients, including supportive care measures like transfusion must account for this unique biological context. Simultaneously, advancements in treatments for other hematologic disorders, such as the innovative use of gene therapy platforms requiring meticulous monitoring for immune-related adverse events, reinforce the critical importance of tailored safety surveillance across all therapeutic modalities in vulnerable populations (13).

Moreover, the vast majority of pediatric patients with malignant solid and hematological tumors require supportive transfusions of red blood cells (RBCs), platelets (PLTs), fresh frozen plasma (FFP), and cryoprecipitate throughout their therapy (14, 15). This repeated antigenic exposure, superimposed on a backdrop of potential immune dysregulation from both the malignancy and cytotoxic therapies, likely creates a unique and heightened risk environment for TRARs (10). Therefore, systematic analyses are critically needed to characterize the incidence, spectrum, and specific risk factors for TRARs within this vulnerable pediatric oncology cohort. In the present study, we retrospectively analyzed the data of TRAR from 2018 to 2024 in Wuhan Children's Hospital (Wuhan Maternal and Child Healthcare Hospital), Tongji Medical College, Huazhong University of Science & Technology. We aimed to determine the clinical characteristics and frequency of TRAR in children with malignant solid and hematological tumors.

## Methods

### Study subjects

From January 2018 to December 2024, hospitalized pediatric patients (<18 years of age) with malignant solid and hematological tumors in Wuhan Children's Hospital (Wuhan Maternal and Child Healthcare Hospital), Tongji Medical College, Huazhong University of Science & Technology were selected. Those who were transfused with RBC, FFP, PLT and cryoprecipitate were enrolled. Children who received albumin, intravenous immunoglobulin, and factor concentrates were not included. This study was performed in line with the principles of the Declaration of Helsinki. Approval was granted by the Ethics Committee of Wuhan Children's Hospital (Wuhan Maternal and Child Healthcare Hospital), Tongji Medical College, Huazhong University of Science & Technology.

During the research period, all blood products were provided by Wuhan Blood Center. RBCs were prepared from random single donors. RBCs were prepared by depleting leukocytes from whole blood using the leukocyte filter, separating the majority of plasma from the resulting leukocyte-depleted whole blood, and adding the RBC additive solution. FFP was prepared by separating plasma from whole blood within 8 h after collection and rapidly freezing it into a solid state. PLTs were obtained directly from single donor's circulating blood via centrifugation using the blood cell separator, while intercepting residual leukocytes through the integrated leukocyte filter. Platelet storage duration was categorized as “fresh” (≤72 h post-collection) or “stored” (>72 h). This threshold was based on common blood bank inventory management protocols and biological considerations related to the progression of the platelet storage lesion, which may influence the product's immunomodulatory potential. Cryoprecipitate was prepared by thawing FFP (derived from whole blood) at controlled temperatures, separating the majority of plasma, and rapidly freezing the remaining cold-insoluble material into a solid state within 1 h. The transfusion indications at our institution are established and implemented accordance with the Chinese guideline for pediatric transfusion.

### Classification of the TRAR

During blood transfusion, nurses recorded the patient's vital signs within the first 15 min after initiation, then at 30-minute intervals until 30 min after transfusion completion. And nurses were required to record and notify the physicians if any new sign(s) and/or symptom(s) occurring during and up to 24 h after transfusion: fever (defined as an increase in temperature ≥1 °C or a temperature at least ≥38 °C, chills, cutaneous manifestations (cyanosis, petechiae, rash, urticaria, pruritus), pain (lumbar, thoracic, headache), hypotension (decrease in systolic blood pressure exceeding 25% of the baseline value), hypertension, dyspnea (chest tightness, tachypnea, wheezing, non-productive cough), vomiting, anxiety and/or agitation, jaundice, red-colored urine (suspicion of hemoglobinuria), oliguria or anuria. The suspected TRAR cases and their management process were received in real time online by the department of blood transfusion to make the initial evaluation. Because our hospital was the member of CHN, the cases were reported through the online reporting platform of the CHN as soon as possible. The final diagnosis and severity definition of the cases were made by CHN experts (5).

### The severity definition the TRAR

Not serious: Physician intervention (e.g., symptomatic treatment) is required, but failure to intervene does not result in permanent impairment of the patient's physical function.

Serious: The patient is hospitalized or hospitalized due to an adverse transfusion reaction; or the patient is disabled or incapacitated due to an adverse transfusion reaction; or medical intervention is necessary to avoid impairment of the patient's physical function.

### Data collection

Cases jointly validated by our department and CHN experts have been enrolled, while other suspected cases have been excluded from our study. The characteristics of the TRAR were collected from the standard transfusion forms, including TRAR type, severity of TRAR, the type, storage duration and donor blood group of blood product, the time from initiation of transfusion to the TRAR, symptom resolution time, the patient's vital signs and clinical intervention. The following clinical data were recorded for each transfused child: age, sex, height, weight, diagnosis, blood group, transfusion history, history of TRAR, allergy history and hospitalization days from electronic medical record system. Allergy history was defined as history of an allergic reaction to medications, food, environmental allergens, or other substances prior to the index transfusion event.

### Statistical analysis

The Kolmogorov–Smirnov test or Shapiro–Wilk test was used to assess normality in continuous variables. Normally distributed data were expressed by mean (standard deviation) and data that were not normally distributed were presented as median and interquartile range (IQR). The independent samples *t*-test or Mann–Whitney *U* test was used to compare the differences of continuous variables. The chi-square test was used to compare the differences of nonparametric data. A *p* value less than 0.05 was considered statistically significant. To account for multiple comparisons, *p* values correction in Table 3 were performed using the Bonferroni method. These analyses were performed using the SPSS 20.0 statistical software (IBM, Chicago, Illinois, USA).

## Results

From January 2018 to December 2024, a total of 9,129 pediatric patients with malignant solid and hematological tumors underwent 34,195 transfusion events of blood products. Of these blood products, 14,033 (41.0%) were RBC, 13,915 (40.7%) were PLT, 5,469 (16.0%) were FFP, and 778 (2.3%) were cryoprecipitate. During the study period, 231 TRAR cases were observed with the total TRAR rate of 680/100,000 transfusions (231/34,195). The TRAR rates of RBC, PLT, FFP and cryoprecipitate were 120/100,000 (17/14,033), 1,250/100,000 (174/13,915), 620/100,000 (34/5,469) and 770/100,000 (6/778), respectively. Of the TRAR cases, 174 (75.3%) were PLT, 34 (14.7%) were FFP, 17 (7.4%) were RBC, and 6 (2.6%) were cryoprecipitate. Of the 231 TRAR cases, 221 were allergic transfusion reactions (ATRs), 8 were febrile non-hemolytic transfusion reactions (FNHTRs) and 2 were other reactions (reactions could not be classified, 1 child had abdominal pain and 1 child had hematuria). The characteristics of the patients were shown in Table 1. The mean age of the patients was 6.08 (3.52) years, of the patients, 156 (67.5%) were male and 95 (41.1%) were O blood group. The main diagnoses were acute lymphoblastic leukemia, neuroblastoma, and acute myeloid leukemia. The patients had blood transfusion history, TRAR history and allergy history were 192, 88 and 74, respectively.

**Table 1:** Demographics of the children with TRAR.

Characteristics	Category/value
Age (year)	6.08 (3.52)
Height (cm)	115.5 (23.9)
Weight (kg)	22.3 (11.9)
Hospitalization days (day)	17 (6–29)
Gender, *n* (%)	
Male	156 (67.5)
Female	75 (32.5)
Diagnosis, *n* (%)	
Acute lymphoblastic leukemia	84 (36.4)
Neuroblastoma	43 (18.6)
Acute myeloid leukemia	35 (15.2)
Aplastic anemia	28 (12.0)
Lymphoma	11 (4.8)
Others	30 (13.0)
Blood group, *n* (%)	
O	95 (41.1)
A	69 (29.9)
B	48 (20.8)
AB	19 (8.2)
Blood transfusion history, *n* (%)	
Yes	192 (83.1)
No	39 (16.9)
TRAR history, *n* (%)	
Yes	88 (38.1)
No	143 (61.9)
Allergy history, *n* (%)	
Yes	74 (32.0)
No	157 (68.0)

As shown in Table 2, there were 194 non-serious cases and 37 serious cases. Of the serious cases, 34 were ATRs, 2 were FNHTRs and 1 was other reaction. The main clinical symptom of non-serious ATR was localized rash accompanied by pruritus. Of the serious ATRs, 14 patients exhibited cutaneous rash accompanied by symptoms affecting respiratory system or gastrointestinal system. Generalized rash accompanied by pruritus was observed in 6 cases. Five cases presented with anaphylactic shock. Five cases presented with facial or limb edema and 4 were drug-unresolved rash. All the body temperature of non-serious FNHTR cases was <39 °C and the body temperature of 2 serious FNHTR cases was >39 °C. Regarding clinical interventions, the vast majority of non-serious cases resolved with corticosteroid or antihistamine therapy. In serious cases, multimodal interventions, including oxygen therapy, epinephrine, normal saline volume expansion and sodium bicarbonate, were typically required in conjunction with corticosteroids and antihistamines for symptom control.

**Table 2:** Clinical symptoms and clinical interventions of the TRAR cases.

Clinical symptoms/interventions	Reaction type	Non-serious	Serious
		*n* = 194	*n* = 37
Clinical symptoms	ATR *n* = 221	Localized rash accompanied by pruritus (166)	Rash + respiratory symptoms (8)
			Rash + gastrointestinal symptoms (6)^a^
			Generalized rash accompanied by pruritus (6)
		Rash + throat dryness or cough (13)	Anaphylactic shock (5)
			Facial or limb edema (5)
			Drug-unresolved rash (4)
		Rash + eyelid or facial edema (8)	
	FNHTR *n* = 8	Fever with body temperature <39 °C (5)	Body temperature >39 °C + chills, nausea/vomiting or diarrhea (2)
		Fever with body temperature <39 °C + chills (1)	
	Others *n* = 2	Abdominal pain (1)	Hematuria (1)
Clinical interventions	ATR *n* = 221	Corticosteroids (113)	Corticosteroids + antihistamines (14)
		Corticosteroids + antihistamines (51)	Corticosteroids + antihistamines + epinephrine + oxygen therapy (5)
		Antihistamines (12)	Corticosteroids + antihistamines + epinephrine (5)
		Initiate close monitoring (9)	Corticosteroids + antihistamines + epinephrine + normal saline volume expansion (5)
		Beta-agonist (2)	
	FNHTR *n* = 8	Initiate close monitoring (1)	Paracetamol + corticosteroids + oxygen therapy (2)
		Paracetamol (2)	
		Corticosteroids (3)	
	Others *n* = 2	Corticosteroids (1)	Corticosteroids + sodium bicarbonate (1)

^a^ Respiratory symptoms including dyspnea, wheezing, tachypnea, laryngeal edema (with pharyngeal discomfort) and cough.

Differences of clinical characteristics between serious group and non-serious group were examined, no statistically significant differences were observed between the two groups of pediatric patients in characteristics, including age, height, weight gender, the time from initiation of transfusion to the TRAR, symptom resolution time, hospitalization days, type of TRAR (ATR vs. non-ATR), blood group (O vs. non-O), PLT (fresh vs. stored), blood transfusion history and TRAR History. There were statistically significant differences in type of blood product (PLT vs. non-PLT), allergy history and prophylactic administration before blood transfusion between the two groups (*p* value = 0.011, 0.048 and 0.000, respectively). After adjustment for multiple comparisons using the Bonferroni correction, prophylactic premedication was significantly associated with the occurrence of serious TRARs (*p* < 0.001), seen in Table 3.

**Table 3:** Differences between serious group and non-serious group.

Characteristics	Total	Non-serious	Serious	*p*	Corrected *p*^e^
	*n* = 231	*n* = 194	*n* = 37		
Age (year)	6.08 (3.52)	6.21 (3.53)	5.44 (3.42)	0.227	>1.000
Height (cm)	115.5 (23.9)	116.5 (23.8)	110.5 (23.7)	0.872	>1.000
Weight (kg)	22.3 (11.9)	22.8 (12.2)	19.3 (10.0)	0.106	>1.000
Gender				0.698	>1.000
Male	156	130	26		
Female	75	64	11		
The time from initiation of transfusion to the TRAR (min)	55 (40–85)	57.5 (40–90)	50 (35–75)	0.284	>1.000
Symptom resolution time (min)	38 (25–70)	35.5 (25–70)	40 (30–90)	0.521	>1.000
Hospitalization days (day)	17 (6–29)	17 (5.7–29)	22 (8.5–33)	0.260	>1.000
Type of TRAR					
Allergic reaction	221	187	34	0.218^a^	>1.000
FNHTR	8	6	2		
Others	2	1	1		
Blood group				0.419^b^	>1.000
O	95	82	13		
A	69	58	11		
B	48	37	11		
AB	19	17	2		
Type of blood product				0.011^c^	0.165
PLT	174	140	34		
FFP	34	32	2		
BRC	17	16	1		
Cryoprecipitate	6	6	0		
PLT^d^				0.623	>1.000
Fresh	132	109	20		
Stored	42	31	14		
Transfusion history				0.282	>1.000
Yes	192	159	33		
No	39	35	4		
History of TRAR				0.482	>1.000
Yes	88	72	16		
No	143	122	21		
Allergy history				0.048	0.720
Yes	74	57	17		
No	157	137	20		
Prophylactic administration before transfusion				0.000	0.000
Yes	104	77	27		
No	127	117	10		

^a^ Difference of allergic reaction and non allergic reaction between serious group and non-serious group.

^b^ Difference of O and non O between serious group and non-serious group.

^c^ Difference of PLT and non PLT between serious group and non-serious group.

^d^ We define PLT collected ≤72 h as fresh PLT and platelets collected >72 h as stored PLT.

^e^ Bonferroni-corrected significance level = 0.0033. *P*-values were compared against this threshold.

## Discussion

This retrospective, single-center study delineates a high rate of TRARs (680/100,000) among pediatric patients with malignant solid and hematological tumors. Our principal findings include a notably high proportion (95.7%) of ATRs, a dominant association with PLT transfusions, and the correlation between the prophylactic premedication and the occurrence of serious TRARs.

Previous studies reported TRARs rate ranged from 540/100,000 to 1,600/1,000,000 among pediatric patients (9, 16, 17). When focusing on single-center studies specifically investigating pediatric oncology or hematology populations, our findings show important parallels and offer more direct context. A retrospective study at a single institution reported an overall TRAR rate highlights the consistent risk in pediatric populations (18). More pertinent to our cohort, a 2022 study by Yanagisawa et al. (19) specifically examined TRARs in pediatric patients with hematological/oncological diseases. 144 of 363 pediatric patients developed ATRs. The TRARs rate was 1,096/225,082 units of blood products from a retrospective observational analysis of adult hematological patients (20). Their work underscores a similarly significant burden, reinforcing our observation that this group faces a distinct risk profile.

Recipient characteristics, blood component type, donor and product quality factors, and administration methods are all recognized risk factors for TRARs (21). Heterogeneity across studies—such as variations in patient demographics (e.g., age distribution, inclusion of critically ill subjects, cohort size), study design parameters (e.g., duration and observation period), and the types and preparation methods of transfused blood products—inevitably leads to divergent reported profiles of TRAR rate and clinical presentation. Consequently, discrepancies exist in the overall TRAR rates, the proportional contribution of different blood components to these reactions, and the distribution of specific TRAR types (e.g., ATR vs. FHNTR) when comparing our findings with those of prior studies. For pediatric patients with malignant solid and hematological tumors, as well as critically ill children, frequent and diverse transfusion exposures are common therapeutic necessities. This repeated antigenic challenge is a recognized contributor to an elevated risk of TRARs (22), which likely accounts, at least in part, for the notably higher reaction rate observed in our specific cohort. Haemovigilance systems are instrumental in the systematic monitoring, reporting, investigation, and analysis of TRARs, facilitating early detection and management (23). However, the definitions, diagnostic criteria, and reporting thresholds are not uniform across different national haemovigilance systems or research protocols (7). These methodological inconsistencies may further contribute to the observed variations in TRAR characteristics and reported rates across different countries and studies.

A single-center study in Atlanta, USA which compared differences of acute TRARs to all blood products between pediatric and adult populations in 2015 (17) and a retrospective research from 9 children's hospitals (9) identified ATRs as exceptionally common, which aligns with our finding. Crucially, the overwhelming predominance of ATRs in our study contrasts with reports from other pediatric contexts, such as a pediatric intensive care unit (16) and a single institutional analyses (18), where FNHTRs are often more common. Overall, ATR and FNHTR are the most frequently reported TRAR among pediatric patients. In the present study, the ATR accounted for 95.7% of all TRARs, a proportion significantly higher than reported in previous literatures (10, 24). This discrepancy can be attributed to several factors: (1) The systematic use of leukocyte-reduced blood components at our center significantly reduces the incidence of FNHTR, effectively unmasking ATRs as the leading identifiable reaction type; (2) ATRs present with distinct and characteristic symptoms, allowing pediatric patients, caregivers, and clinical staff to make rapid and accurate assessments. Additionally, healthcare providers have developed a well-established understanding of the strong causal relationship between ATR and blood transfusion; (3) pediatric patients with malignant solid and hematological tumors exhibit heightened baseline susceptibility to disease-related pyrexia. Such febrile episodes may obscure FNHFRs, potentially leading clinicians to perceive a weaker causal association between fever and blood transfusion, thereby contributing to underreporting; (4) some cases of ATR with co-occurring fever were only reported as ATRs; (5) inadequate temperature monitoring during blood transfusion-frequent protocol deviations and overreliance on subjective symptom reporting-led to undocumented low-grade fevers; (6) frequent transfusions, combined with the disease-related and therapy-induced immunomodulatory state in pediatric oncology patients, creates a milieu that predisposes them predominantly to allergic-type hypersensitivity reactions over febrile responses.

The risk of ATRs is notably higher with FFP and PLT transfusions than with RBC transfusions (25). Previous studies in pediatric populations (9, 26) from the United States reported ATR rates ranging from 302 to 624 per 100,000 PLT transfusions. In contrast, our study observed a substantially higher ATR rate of 1,250 per 100,000 PLT transfusions. This elevated incidence may be partly attributed to the exclusive use of apheresis platelet concentrates in our cohort. The high concentrations of allogeneic plasma proteins containing in apheresis platelet concentrates have been associated with an increased risk of TRARs, including ATRs, compared to other blood components and preparation methods (27).

The high rate of TRARs in pediatric patients with malignant solid and hematological tumors necessitates a detailed characterization of their clinical features. In current practice, the recognition and management of ATR and FNHFR are timely, with favorable prognoses for most cases. The majority of these reactions are mild and transient, resolving spontaneously or with symptomatic pharmacologic intervention. However, severe reactions, though rare, present diagnostic challenges due to their complex clinical presentations. Moreover, domestic reports detailing the clinical profiles of such severe reactions remain limited. Findings revealed that the majority of TRARs were mild, while approximately 16.0% (37/231) were classified as serious cases. Among serious cases, 91.9% (34/37) were ATRs, and 5.4% (2/37) were FNHFRs. Beyond common skin mucosal symptoms, respiratory or gastrointestinal manifestations occurred in 41.2% (14/34) of serious ATRs. All of 5 cases of anaphylactic shock resolved with prompt clinical resuscitation, with no transfusion related fatalities.

Unlike international studies (28, 29), our research recorded no cases of transfusion associated circulatory overload (TACO) or transfusion related acute lung injury (TRALI). Lake of diagnostic criteria to pediatric patients and insufficient clinical awareness are main reasons of difficulties in diagnosis of TACO and TRALI. Atypical presentations further complicated diagnosis, particularly in young pediatric patients with limited verbal expression. In such cases, clinicians should monitor vital signs (heart rate, respiratory rate/depth, SpO_2_, BP fluctuations), inspect skin changes (remove clothing to assess for rashes/edema) and utilize diagnostics (ECG, cardiopulmonary imaging). Early recognition of severe reactions in children is critical. Prompt diagnosis facilitates targeted resuscitation, improving clinical outcomes (30). In severe TRAR, clinicians start corticosteroids for all patients—even without confirmed ATR. Most TRARs are assumed to be allergic, even without rash. Corticosteroids are used for their anti-allergic and anti-inflammatory effects in critical cases (31).

Among serious ATRs, epinephrine was administered to 44.1% (14/34) of cases. The 2023 British Society for Haematology (BSH) Guidelines for Acute Transfusion Reactions (32) stress that initial treatment should focus on relieving symptoms and signs, rather than waiting for a definitive ATR diagnosis. For life-threatening reactions, immediate empirical treatment is critical. UK Resuscitation Council guidelines recommend intramuscular epinephrine as the first-line treatment for anaphylaxis. Similarly, the European Academy of Allergology and Clinical Immunology (EAACI) Pediatric Anaphylaxis Guidelines (33) clearly state that intramuscular epinephrine is the first-line therapy for severe ATRs in children. Glucocorticoids are not recommended as the primary intervention due to their delayed onset of action. All supportive treatments-including fluid resuscitation, bronchodilators, antihistamines, or glucocorticoids-are secondary to epinephrine. Nevertheless, many clinicians still have persistent knowledge gaps about anaphylaxis severity and epinephrine indications (34), wrongly reserving it only for cases with clear cardiorespiratory compromise or refractory hypotension. In the present study, 2 serious FNHTR cases presented with fever (>39 °C), chills, nausea/vomiting or diarrhea. Symptoms resolved after symptomatic treatment with paracetamol combined with corticosteroids. Serious FNHTR typically manifests as the temperature elevation ≥1 °C accompanied by systemic symptoms, predominantly gastrointestinal manifestations. Differentiation serious FNHTR from hemolytic reaction or bacterial contamination requires comprehensive evaluation correlating clinical features-such as rash, back pain, hematuria, or hypotension-with microbiological tests (e.g., bacterial cultures) (35). For pediatric patients with malignant solid and hematological tumors, fever may arise from the underlying malignancy, bone marrow suppression related infections, or blood transfusion. Consequently, attributing post-transfusion fever definitively to transfusion remains challenging. Adherence to blood transfusion protocols, including pre-transfusion temperature assessment, transfusion blood only if normothermic, continuous temperature monitoring during blood transfusion, is critical for early TRAR detection and prompt management.

The time from transfusion start to TRAR onset is not well documented in children. While one adult study reported an average onset of approximately 100 min, with earlier onset in severe cases (36), our pediatric study found a median time of 55 min, with no significant difference between serious and non-serious reactions. Previous pediatric studies (24, 37) reported median onset times ranging from 30 min to 2 h, with no clear distinction by reaction severity. Although study designs vary and direct comparisons are limited, all evidence suggests a broad window for TRAR occurrence. Therefore, continuous monitoring throughout the entire transfusion process is critical for early detection in pediatric patients.

In our study, serious TRARs were, to some extent, associated with children with allergy history. In the univariate analysis, allergy history was identified as a potentially relevant factor, recipient related factors were more associated with ATR (38, 39). Essentially, ATR is immediate hypersensitivity reaction, which is closely related to the patient's blood transfusion history and allergy history (40). Blood products contain sensitizing factors (primarily plasma proteins). Patients with malignant solid and hematological tumors require frequent blood transfusions. The repeated exposure to these sensitizing factors makes the body more prone to actively develop antibodies or bioactive mediators associated with allergic responses. Upon subsequent transfusions, patients are more susceptible to experiencing ATRs. Moreover, patients with allergy history often possess an allergic predisposition. Their allergy effector cells remain in an activated state and are more likely to trigger allergic reactions when exposed to sensitizing factors in blood products. However, allergy history was not a statistically significant independent risk factor after robust adjustment, suggesting it requires further investigation in larger, prospective studies.

For patients with previous mild ATRs, there is no evidence to support the routine use of prophylaxis administration with antihistamines or glucocorticoid before blood transfusion. Only low-grade evidence exists to recommend pre-transfusion antihistamines for those with severe ATRs (3). However, in current clinical practice, clinicians still widely believe that prophylactic administration before blood transfusion helps to reduce TRARs (both ATR and FNHTR). Therefore, prophylactic administration before blood transfusion has been used to prevent TRARs in adults and children with its uncertain efficacy (41, 42). In our study, TRARS occurred in 104 pediatric patients despite prophylactic administration with antihistamines or glucocorticoid before blood transfusion. Moreover, the proportion of children receiving such pretreatment was significantly higher in the serious group than in the non-serious group. According to previous studies, prophylactic administration before blood transfusion was only necessary when children receiving transfusion of the same blood component products after a prior ATR (43). Dexamethasone, a commonly used long-acting glucocorticoid, exhibits potent anti-allergic and immunosuppressive effects. It functions by binding to receptor complexes and DNA to influence gene expression, taking at least 1 h to take effect. The main time from initiation of transfusion to the TRAR 55 min in our study. Thus, the timing of medication administration may be one of the reasons why prophylactic administration before blood transfusion fails to reduce TRARs.

Additionally, in our study, all PLTs were apheresis PLTs. Compared with other blood products, PLT presented as a factor that showed the association with higher rate and severity of ATR in univariate analysis. The use of PLT additive solutions decreases the rates of ATR and FNHTR, and reduces severity of ATR and FNHTR (44). However, the infectious risk and reduced PLT concentrations (27) associated with PLT additive solutions may compromise transfusion outcomes in pediatric patients with malignant solid and hematological tumors. Furthermore, the association between PLT transfusion and serious TRARs became non-significant after performing a *p*-value correction. Therefore, further researches concerning the optimal PLT product categories for pediatric patients with malignant solid and hematological tumors were required.

### Limitations and future research directions

The retrospective nature of data collection from a single institution limits the generalizability of our findings. The results may reflect the specific patient demographics, clinical practices, and hemovigilance reporting standards of our center and region, and may not be directly applicable to other healthcare settings. The potential selection bias and the inability to establish causal relationships or assess the long-term outcomes of different intervention strategies. The reliance on clinical documentation also introduces the potential for underreporting or inconsistent documentation, particularly of mild TRARs (e.g., low-grade fever, localized rash) in younger children with limited verbal ability. The study did not control for timing/dosing of premedication (e.g., dexamethasone's delayed onset), potentially confounding its observed inefficacy in preventing serious TRARs. Furthermore, our study did not capture data on whether transfusions were administered as part of planned therapy or in an emergency setting. This contextual factor could potentially influence both the risk and the timely recognition of TRARs, particularly during periods with reduced clinical staffing, and merits investigation.

Large-scale, prospective studies involving multiple pediatric oncology centers across diverse geographical locations are essential. Such studies would employ standardized, active surveillance protocols to minimize reporting bias, and are necessary to validate our reported incidence, establish generalizable rates and risk profiles for TRARs in this vulnerable population. Prospective studies are needed to investigate the association between specific clinical settings (e.g., major surgery requiring massive transfusion support) and TRAR risk. Future studies should aim to incorporate additional contextual variables, such as the elective vs. emergency nature of the transfusion and the shift timing (e.g., day vs. night), to better understand their potential role as risk modifiers for the severity or detection of TRARs.

## Conclusion

This retrospective study highlights a high TRAR rate (680/100,000) in pediatric patients with malignant solid and hematological tumors, predominantly linked to PLT transfusions and manifesting as ATRs (95.7%). Serious TRARs often involved multisystem involvement, necessitating prompt epinephrine-based intervention despite prevalent corticosteroid misuse. Prophylactic administration before blood transfusion correlated with serious reactions. Rigorous vital sign tracking before, during and after blood transfusion is important in pediatric patients with malignant solid and hematological tumors, especially for PLT transfusions.

Further prospective multicenter studies for evidence-based anaphylaxis management, reconsideration of routine premedication and validate risk factors and optimize PLT product selection for pediatric patients with malignant solid and hematological tumors are necessary.

## Data Availability

The raw data supporting the conclusions of this article will be made available by the authors, without undue reservation.
